# First introduction of pandemic influenza A/H1N1 and detection of respiratory viruses in pediatric patients in Central African Republic

**DOI:** 10.1186/1743-422X-10-49

**Published:** 2013-02-08

**Authors:** Emmanuel Nakouné, Vianney Tricou, Alexandre Manirakiza, Francis Komoyo, Benjamin Selekon, Jean Chrysostome Gody, Kathleen Victoir, Philippe Buchy, Mirdad Kazanji

**Affiliations:** 1 National Influenza Centre, Institut Pasteur de Bangui, Bangui, Central African Republic; 2 Virology department, Institut Pasteur de Bangui, Bangui, Central African Republic; 3 Epidemiology Unit, Institut Pasteur de Bangui, Bangui, Central African Republic; 4 Complexe Pédiatrique de Bangui, Bangui, Central African Republic; 5 International Division, Institut Pasteur, Paris, France; 6 Virology Unit, Institut Pasteur du Cambodge, Phnom Penh, Cambodia

**Keywords:** Molecular diagnosis, Acute respiratory illness, Pandemic influenza A/H1N1 2009, Influenza B, Respiratory syncytial virus, Parainfluenza virus, Pediatric patients

## Abstract

**Background:**

Acute viral respiratory illnesses in children in sub-Saharan Africa have received relatively little attention, although they are much more frequent causes of morbidity and mortality than in developed countries. Active surveillance is essential to identify the causative agents and to improve clinical management, especially in the context of possible circulation of pandemic viruses.

**Findings:**

A prospective study was conducted in the Central African Republic (CAR) between January and December 2010 among infants and children aged 0–15 years attending sentinel sites for influenza-like illness or acute respiratory illness. Nasopharyngeal swabs were collected, and one-step real-time and multiplex reverse transcription-polymerase chain reaction were used to detect respiratory viruses. Respiratory viruses were detected in 49 of the 329 (14.9%) nasopharyngeal samples: 29 (8.8%) contained influenza viruses (5 (1.5%) had pandemic influenza A/H1N1 virus and 24 (7.3%) had influenza B viruses), 11 (3.3%) contained parainfluenza viruses types 1 and 3 and 9 (2.7%) contained human respiratory syncytial virus. Most cases were detected during the rainy season in the CAR. Analysis of the amplicon sequences confirmed the identity of each detected virus.

**Conclusions:**

The influenza surveillance system in the CAR has provided valuable data on the seasonality of influenza and the circulation of other respiratory viruses. Our network could therefore play a valuable role in the prevention and control of influenza epidemics in the CAR.

## Finding

Although acute respiratory illness is a major cause of morbidity and mortality among children in sub-Saharan Africa, it has received relatively little attention [1]. This is unfortunate, as underlying diseases such as AIDS, malaria and tuberculosis, which are highly prevalent in the region, can worsen such illnesses [2]. The respiratory viruses known to cause acute illness include human respiratory syncytial virus (HRSV), human parainfluenza virus (PIV), human metapneumovirus and influenza viruses [3-5]. Until recently, the burden of influenza and influenza-like illness in Africa was considered to be negligible [6], mainly because of the lack of confirmation assays. Reports from Cameroon and Senegal, however, show that influenza viruses are actively circulating and may be causing regular epidemics [7,8].

A clear picture of the contribution of each pathogen to acute respiratory illness is needed in order to improve prevention and clinical management and consequently to reduce the burden of disease. The emergence of the novel influenza A/H1N1 of swine origin in Mexico in April 2009 and its rapid spread worldwide, causing a global pandemic, led the health authorities of the Central African Republic (CAR) to collaborate with the World Health Organization in strengthening biological surveillance of acute respiratory illness.

The aim of the study reported here was to determine the circulation of 2009 pandemic influenza A/H1N1 virus (H1N1pdm09) by molecular methods and to identify the causative viruses, the incidence and the clinical features of acute respiratory illness among infants and young children at sentinel sites in Bangui and three rural areas.

**Figure 1:**
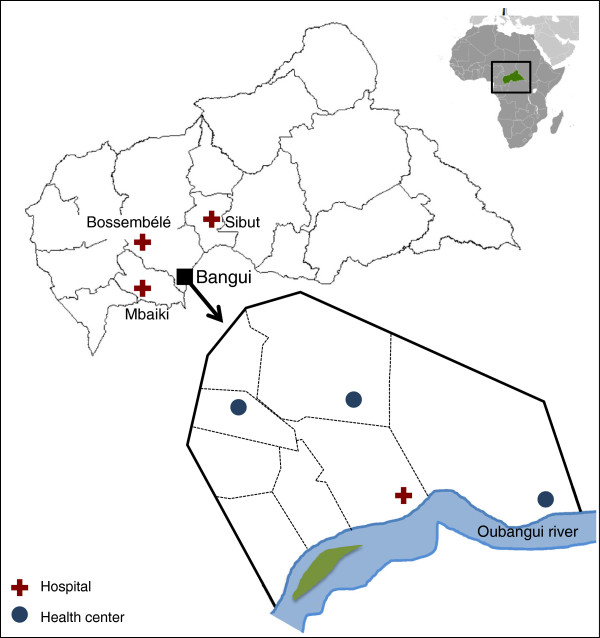
Locations of sentinel sites for surveillance of influenza and respiratory viruses in the Central African Republic.

**Figure 2:**
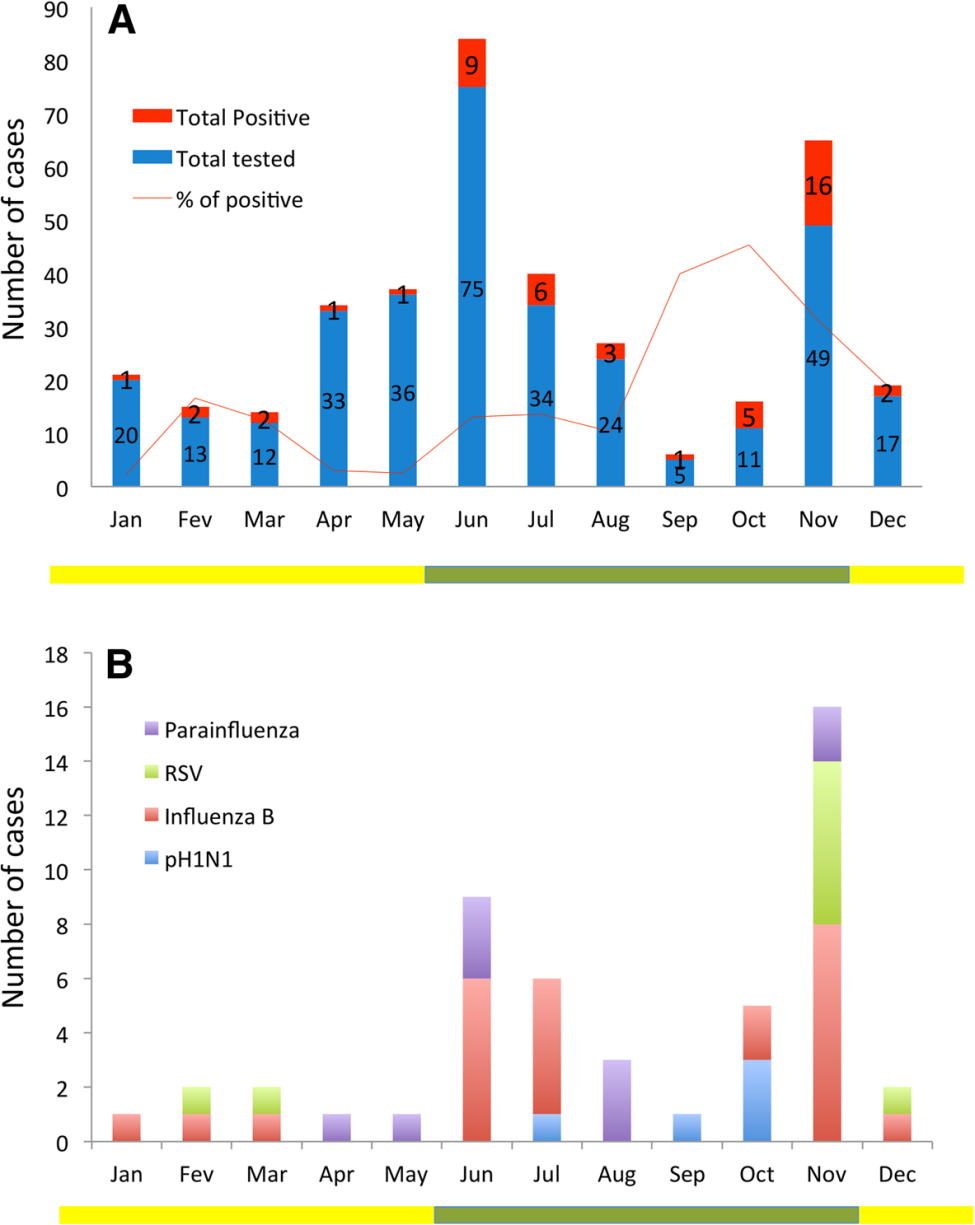
**Seasonal patterns of influenza and other respiratory viruses in the Central African Republic.** (**A**) monthly numbers of infants and children included in the study with detection rate of total respiratory viruses. (**B**) monthly numbers of cases of influenza and other respiratory viral illness. The dry season is December–May (yellow line), and the rainy season is June–November (green line).

All infants and children aged between 0–15 years who attended sentinel sites in Bangui and three rural areas (Figure 1) for influenza-like illness (ILI) or severe acute respiratory illness between January and December 2010 were included in the study (Figure 2A). The World Health Organization definitions were used for ILI (sudden onset of fever of > 38°C and cough or sore throat in the absence of other diagnoses) and severe acute respiratory illness (ILI symptoms *and* shortness of breath or difficulty in breathing *and* requiring hospital admission). The study protocol was approved by the National Ethics Committee of the CAR. Individual written informed consent was sought from the parents or guardians of all participants. 

Nasopharyngeal samples were collected from 329 infants and children and within 48 hrs at 4°C to the National Influenza Centre by using rayon-budded swabs with virus transport medium pre-impregnated sponge (Virocult, Medical Wire & Equipment, UK).

RNA was extracted with a QIAmp RNA mini kit (Qiagen) according to the manufacturer’s instructions. Influenza A viruses were detected with a previously described assay targeting the conserved matrix gene for universal detection of these viruses [9], and H1N1pdm09 virus was identified with a specific one-step real-time reverse transcription-polymerase chain reaction (RT-PCR) assay (designed by the National Influenza Centre of northern France, Institut Pasteur, Paris; primers and probe available upon request at grippe@pasteur.fr). All specimens were also tested for other respiratory viruses in two previously described multiplex semi-nested RT-PCR assays for detecting influenza A and B viruses, HRSV, human metapneumovirus and PIV types 1, 2, 3 and 4 [10,11]. All assays were performed on an ABI 7500 platform (Applied Biosystems, Foster City, California, USA) with the SuperScript III Platinum One-step Quantitative RT-PCR System (Invitrogen, Carlsbad, California, USA). A specimen was considered positive if the signal curve crossed the threshold line within 40 cycles. The assay limit of detection for pandemic H1N1pdm09 influenza virus is of order of magnitude of 10 copies/μL of initial sample [9]. The assay limit of detection for influenza A and B viruses, HRSV, human metapneumovirus and PIV-3 is of order of magnitude of 10 copies/μL [10]. For PIV-4, the assay limit of detection is of order of magnitude of 100 copies copies/μL, and for PIV-1 and −2, 1000 copies/μL [10]. After amplification, the PCR products were purified and sent to GATC Biotech (Konstanz, Germany) for sequencing.

Student’s *t* test and the Pearson chi-squared test were used to assess intergroup differences. Statistical analyses were performed with EpiInfo software (V 3.5.1 CDC). A test was considered significant when the *p* value was < 0.05. The newly obtained sequences were analysed and compared with sequences available in GenBank.

## Results and discussion

Of the 329 patients included in the study, 49 (14.8%) tested positive for respiratory viruses (Table 1). Of these, five (1.5%) were positive for H1N1pdm09, 23 (6.9%) for influenza B, 10 (3.0%) for HRSV and 11 (3.3%) for PIV-1 and −3 (Table 1 and Figure 2B). The average age of positive patients was 41 months (range, 1 month to 9 years), with no difference in the age or gender distribution. All samples were negative for human metapneumovirus.

**Table 1:** Respiratory viruses identified in swab samples from 329 patients with influenza-like illness in Bangui, Central African Republic, by age group

**Age group**	**Number of cases**	**Respiratory virus detected (N positive/N tested (%))**					
		**H1N1pdm09**	**RSV**	**PIV 1**	**PIV 3**	**Influenza B**	**All**
0-6 m	61	0/61	3/61 (4.9)	1/61 (1.6)	2/61 (3.3)	5/61 (8.2)	11/61 (18.0)
6 m-1y	73	0/73	2/73 (2.7)	2/73 (2.7)	2/73 (2.7)	7/73 (9.6)	13/73 (17.8)
1-2y	123	2/123 (1.6)	2/123 (1.6)	1/123 (0.8)	2/123 (1.6)	8/123 (6.5)	15/123 (12.2)
2-5y	49	2/49 (4.0)	2/49 (4.0)	0/49	1/49 (2.0)	3/49 (6.1)	8/49 (16.3)
6-15y	23	1/23 (4.3)	1/23 (4.3)	0/23	0/23	0/23	2/23 (8.6)
All	329	5/329 (1.5)	10/329 (3.0)	4/329 (1.2)	7/329 (2.1)	23/329 (6.9)	49/329 (14.8)

m, months; y, years, H1N1pdm09, pandemic influenza A/H1N1 2009; RSV, respiratory syncytial virus; PIV, parainfluenza virus.

The first case of H1N1pdm09 was detected in the CAR on 26 July 2010 in a child aged 36 months, followed by a second case on 11 September in an infant aged 16 months, and three cases were found in young children on 4, 11 and 22 October (Figure 2B). Of the five children, only one was admitted to intensive care for respiratory distress; no deaths were recorded. A 253-bp fragment of the haemagglutinin gene of the five H1N1pdm09 strains [accession numbers: CY092425, CY092426, CY092427, CY092428, CY092429] showed high sequence similarity (99–100%) to influenza A/California/04/2009 (H1N1) (data not shown).

The 23 specimens positive for influenza B represented 82.1% of the influenza viruses detected. Ten of the patients were hospitalized; one infant aged 9 months died 2 days after admission to intensive care with a clinical picture of severe acute respiratory illness. Influenza B virus was implicated in respiratory infections throughout the year, with two peaks: a first (11 positive samples) at the beginning of the rainy season and a second (8 positive samples) in November at the end of the rainy season (Figure 2B).

Partial genome analysis of nine amplified samples of influenza B virus [accession numbers: HE803088, HE803089, HE803090, HE803091, HE803092, HE803093, HE803094, HE803095] demonstrated high nucleotide similarity (98%) to vaccine strain B/Victoria/02/1987, except for one that belonged to the Yamagata lineage (data not shown).

HRSV was detected in 10 patients (Table 1) with a median age of 28 months, mainly in November (Figure 2B). One infant aged 10 months died 1 day after admission to intensive care with a clinical picture of fever, cough, rhinitis, bronchiolitis, dyspnoea and myalgia. Fever and rhinitis were recorded in 9 of the 10 patients, and all had cough. No viral co-infections were reported. Analysis of the nucleotide sequences of six isolates [accession numbers: HE803082, HE803083, HE803084, HE803085, HE803086, HE803087] showed sequence similarity to both genotypes A and B.

PIV-3 was detected in seven patients (median age, 16 months; range, 5–48 months) and PIV-1 in four infants (median age, 11 months; range, 3–24 months) between April and November 2010 (Figure 2B).

Respiratory viruses were found in 14.8% of the 329 collected samples, showing the presence of H1N1pdm09 infections in the CAR for the first time. We found that influenza B, PIV-1, PIV-3 and HRSV were also involved in ILI in the country. Most of the cases were detected during the rainy season.

H1N1pdm09 infection in the CAR was first described in July 2010. In other African countries, the virus was shown to have been introduced by travellers [12-14], but its source in the CAR has not been elucidated. All four cases detected were indigenous, with no history of travel or contact with a person returning from a country with declared cases. Extensive investigations of contacts of the confirmed cases did not reveal any other cases, suggesting low dissemination of H1N1pdm09 in the country. The clinical picture of H1N1pdm09 infection was similar to that of seasonal influenza circulating before the pandemic. This is in accordance with studies showing relatively low transmissibility and severity of H1N1pdm09 [15,16]. Consequently, H1N1pdm09 did not appear to have had a significant public health impact in this area of the world.

In the present study, influenza B virus was the most commonly detected respiratory virus (n=23), whereas in similar studies in Africa HRSV was the most frequent causative virus of ILI [17-19]. Influenza B virus caused respiratory infections throughout the year, with two peaks: at the beginning of the rainy season (June–July) and at the end of the rainy season (November). Antigenically and genetically distinct lineages of influenza B virus, influenza B/Victoria and B/Yamagata viruses have circulated in the CAR [20].

HRSV is a major cause of ILI among infants and children worldwide [21] and is the most frequently detected respiratory virus in both developed and developing countries [17-19,22-26]. In this study, HRSV was found in only 2.7% of the samples and represented 18.3% of the viruses detected. The difference from other studies might be due to a different epidemiology of HRSV in the CAR, a landlocked country in central Africa, or to different inclusion criteria or detection techniques. Most of HRSV cases detected in our study occurred between November and February, corresponding to the dry season in the CAR.

Another interesting finding was the relatively low prevalence of respiratory viruses in the children with ILI, which were present in only 14.9% of samples; in similar studies, as many as 50% of samples contained respiratory viruses [17-19,22-26]. In the CAR, vaccines against *Streptococcus pneumoniae* and *Haemophilus influenzae* were introduced in the enlarged programme of vaccination only recently (*H. influenzae* in September 2008 and *S. pneumoniae* in July 2011). In a study in Bangui in 1995, these bacteria were found in hundreds of ill children aged less than 5 years [27]. It is therefore likely that these bacteria are still very common or even the main causes of respiratory infections in the CAR, as in other countries before the introduction of vaccines, when most severe respiratory infections were due to bacterial infections, and *S. pneumoniae* and *H. influenzae* were the commonest bacterial causes [28-30]. This may explain, at least partly, the differences between our results and those of other studies. It is also possible that viruses other than these we looked for were involved, such as human rhinovirus, human bocavirus, human coronavirus or adenovirus.

The main limitation of our study is the limited sample size, which prevented us from investigating associations between clinical outcome and viral etiology. Another weakness is a bias toward younger patients, so that we could not assess whether a particular age group is at greater risk for a specific infection. Owing to the design of the study, it is also difficult to determine prevalence from the results. Therefore, further studies are needed to evaluate the burden of all respiratory viruses infections in the general population of the CAR.

Our study does highlight the importance of the clinical, epidemiological and virological network for influenza surveillance in the CAR [31,32].

We reported here the first data on the etiology of ILI in the CAR. This will help central African clinicians to provide better care and treatment for patients presenting with ILI, including better use of antibiotics. Further studies with more patients are needed to confirm the burden of viral respiratory diseases in the CAR. Collection of samples from healthy control children may also enable comment on virus detection and disease association. Another suggestion for future studies is to collect data about underlying health status of children as risk factors such as HIV infection, malaria, malnutrition, etc. might have an impact on the acute respiratory infections [33,34]. The temporal patterns detected should be assessed over many years in order to identify long-term seasonal patterns.

## Abbreviations

CAR: Central African Republic; ILI: Influenza-like illness; HRSV: Human respiratory syncytial virus; PIV: Parainfluenza viruses.

## Competing interests

The authors declare that they have no competing interests.

## Authors’ contributions

FK and BS carried out the serological and molecular studies. AM compiled the epidemiological data. EN, VT and MK participated in the design of study, the analysis and the interpretation of the data and drafted the manuscript. AM, JCG, KV and PB participated in the analysis and the interpretation of the data. All authors read and approved the final version of the manuscript.
